# Development of a PCR-based assay for detection of Chinese mink tissue in meat products based on the mitochondrial DNA cytochrome-*b* gene

**DOI:** 10.1080/23802359.2018.1532828

**Published:** 2019-07-26

**Authors:** Jinxia Ai, Liyuan Sun, Lijun Gao, Wei Xia, Mingcheng Li, Siqi Duan, Kun Chen, Guangxin Yuan, Dan Li

**Affiliations:** a School of Laboratory Medicine, Beihua University, Jilin, China;; b School of Pharmacy, Beihua University, Jilin, China;; c Jilin Leining Scientific Service Co. Ltd for Detection of Drug and Food, Jilin, China

**Keywords:** Chinese mink, species authentication, meat product, cytochrome *b*

## Abstract

Species authentication of meat product origins has become an important subject for ensuring the health of consumers. Based on the cytochrome *b* gene, we developed a PCR-based assay kit for identification of Chinese mink tissues from 10 animal species in meat products, and to evaluate its quality indices including specificity, stability, sensitivity, and repeatability. Kits were made up of DNA extraction and PCR amplification systems based on species-specific, and universal primers. The reference meat mixtures and commercial samples were extracted by the kit and PCR technique was performed to identify the species of mink authenticity. The kit was effective after 20 repeated freeze–thaw cycles and it could be stored at –20 °C for 1 year. The sensitivity showed that a concentration as low as 0.1 ng/μL still can amplify the target band. The specificity test confirmed that the kit was 100% specific. The kit proved to be effective, stable, and reliable for extraction of efficient contents of the genomic DNA and routine analysis of Chinese mink source composition from meat products.

## Introduction

According to data published in the Global Food Policy Report of 2016 released by the International Food Policy study, *per capita* meat consumption in China was 59 kg/year. Therefore, food safety in meat, and meat products, is becoming increasingly important. Of all the food safety problems, the most important is food fraud, which is adulteration or substitution of expensive meat with an inferior meat. Recent years have seen an increase in the number of fraudulent cases involving deadly adulterants and legal prosecution for food fraud (Van Ruth et al. 2017).

The species of *Martes zibellina* and *Mustela vison* are the main species of Chinese mink for fur animals, and mink has been in growing demand for fur coat manufacture in China. Actually, mink furs are obtained to produce coats and animal carcasses are disposed of; however, some meat wholesalers are committed to illegal adulteration of their meat products for extra profit. The adverse effect of red meat consumption under the risk of meat fraud is a major public health concern, especially in developing countries in which there are clear trends towards increased consumption (Lopez-Oceja et al. 2017). The quality and authenticity of meat have attracted attention from consumers and relevant government authorities. Therefore, the establishment of a rapid, sensitive, specific detection method to achieve effective supervision has become an urgent problem in the field of food safety (Alikord et al. 2017).

PCR-based molecular biology technology has become the mainstream method for assay of meat-based ingredients in food identification technology (Irwin et al. 1991). Our previous work has developed a new species-specific PCR method identifying mink heart. A pair of primers was designed based on the cytochrome *b* (*Cyt b*) gene in mitochondrial DNA (mt DNA) to amply the specific fragment (Li et al. 2014). The purpose of the study was to develop a DNA detection kit used for determination of the species of mink origin of meat components, and evaluate its parameters with mixed pure meat samples and common consumption meat products.

## Materials and methods

### Sample collection

The standard reference meat components including beef (QC-AN-001), mutton (QC-AN-002), pork (QC-AN-003), horse (QC-AN-004), rabbit (QC-AN-005), deer (QC-AN-006), duck (QC-AN-007), chicken (QC-AN-011), fox (QC-AN-013), and martes (QC-AN-014) were purchased from the National Center for Standard Reference Material, China. They were used as reference samples in the study and certified by Jilin Food Research Institute, National Institute for Food and Drug Control, China. A total number of 11 commercial samples consisting of beef, mutton, pork, chicken, duck, deer, horse, rabbit, and fox in addition to dog and donkey meat were purchased from a local supermarket and from randomly selected farmers’ markets in the region. All animals’ tissues in the study were carried out to comply with the Animal Research: Reporting In Vivo Experiments (ARRIVE) guidelines (Ethical approval number: Protocol Number 2015-08-15).

### Extraction of genomic DNA from meat products with the kit

One gram of each fresh sample was cut into small pieces, and dry samples (mass ≥0.1 g) were washed, air-dried, cut into pieces, and stored in a 2-mL tube. The extraction protocol was done as previously described (Zhang et al. 2018). The precipitate was dried at room temperature, dissolved in 100 μL TE solution, and stored at –20 °C.

### PCR reaction

1.0 μL of each sample of genomic DNA extracts, and the positive and negative control were added into PCR amplification systems tubes. The total volume was adjusted to 30 μL with sterilized water. The PCR conditions were performed as previously described (Zhang et al. 2018).

### Evaluation of specificity, sensitivity, and stability on the kit

Batches of 50 kits were prepared in advance and stored at –20 °C. Two kits were randomly selected to testify specificity, sensitivity, and stability. Some 1 g of authentic sample, the content of each sample of which genomic DNA was determined by ultraviolet spectrophotometer and then diluted used for PCR amplification.

## Results

### Evaluation parameters

**Figure 1.**
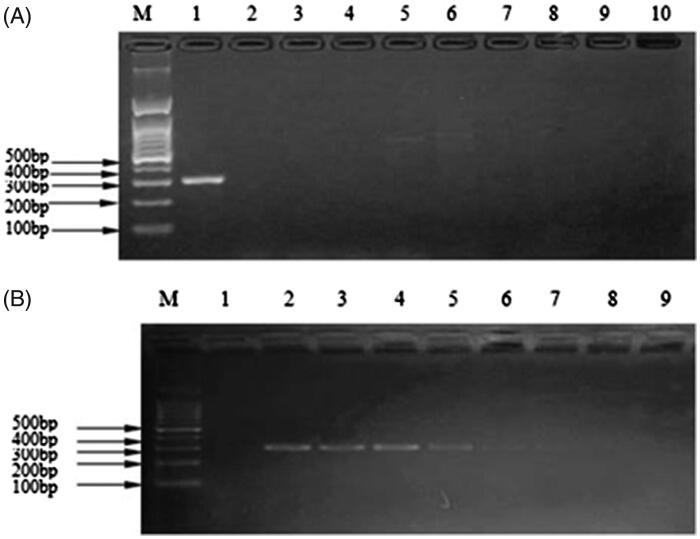
Evaluation of specificity and sensitivity on the kit. (A) Agarose gel electrophoretogram of specificity experiment for *Martes zibellina* and *Mustela vison* tissues mixture detected by the kit. M: DNA Marker; 1: *Martes zibellina* and *Mustela vison tissues*; 2: beef; 3: lamb; 4: pork; 5: donkey; 6: deer; 7: chicken; 8: duck; 9: fox; 10: negative control. (B) Agarose gel electrophoretogram for the sensibility experiment of the kit. M: Marker; 1: negative control; 2: 100 ng/μL; 3: 10 ng/μL; 4: 1 ng/μL; 5: 0.1 ng/μL; 6: 0.01 ng/μL; 7: 0.001 ng/μL; 8: 0.0001 ng/μL; 9: 0.00001 ng/μL.

After PCR amplification, only mink meat had specific binding sites with the specific primers with amplification products of about 200–300 bp consistent with the positive control, however, the negative control and others did not have amplification bands (Figure 1(a)). The kit’s specificity was 100% (Figure 1(b)). The results of sensitivity showed that sample as low as 0.1 ng/μL still can amplify the target band between 200 and 300 bp. The kit was repeatedly frozen and thawed 5, 10, 15, and 20 times and yielded consistent results, amplifying target bands between 200 and 300 bp of the extraction of genomic DNA, showing that the kit had good stability. The kit was stored at –20 °C for one year, and amplification products still presented the distinct target band of 200–300 bp consistent with the positive control, indicating its validity was at least 1 year.

### Reference mixtures and commercial samples

**Figure 2.**
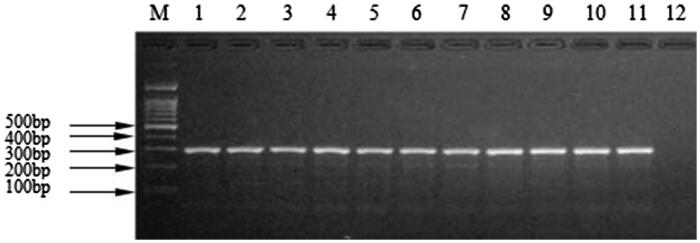
Agarose gel electrophoretogram for meat mixture of *Martes zibellina* and *Mustela vison* tissues detected by the kit. M: DNA Marker; 1: *Martes zibellina* and *Mustela vison*; 2: Beef + *Martes zibellina* and *Mustela vison*; 3: Lamb + *Martes zibellina* and *Mustela vison*; 4: Pork + *Martes zibellina* and *Mustela vison*; 5: Donkey + *Martes zibellina* and *Mustela vison*; 6: Deer + *Martes zibellina* and *Mustela vison*; 7: Chicken + *Martes zibellina* and *Mustela vison*; 8: Duck + *Martes zibellina* and *Mustela vison*; 9: Dog + *Martes zibellina* and *Mustela vison*; 10: Rabbit + *Martes zibellina* and *Mustela vison*; 11: Fox + *Martes zibellina* and *Mustela vison;* 12: negative control.

The 10 reference meat mixtures prepared for the study were analyzed and the species determination for all mixtures was identical in accordance with the expected data for each sample. All of the commercial meat products, including beef, mutton, pork, chicken, duck, venison, dog, rabbit, donkey, and fox, when mixed with 10% by mass of mink meat were presented as positive (Figure 2). The findings demonstrated that the kit can be used for detection of commercially available meat products containing all reported additives on product labels under different processing regimes including high temperatures, high pressures, fermentation, and fume smoking, presenting the same results in all conditions mentioned.

## Discussion

Meat products are an important nutritious substance that is closely related to people’s lifestyles and health: reports of high-priced meat with cheap meat or toxic and harmful meat are often present. Actually, food fraud is much more prevalent than most Chinese consumers realize. Some unscrupulous enterprises and individuals bought these meat, sold it to the black-market factories made of mutton roll, meatballs, bacon, ham, and other foods, which cannot be identified after processing with beef and mutton by the naked eye.

PCR-based DNA detection technology has gradually become the core method in identification of meat species: the mitochondrial Cyt b, 12S rRNA, and D-loop regions are the target sequences (Castresana 2001; Xin et al. 2009). Some researchers reported the use of the PCR method for the detection of horse, donkey, deer, and camel components (Yacoub and Sadek 2017). Multiplex PCR technology is also widely used in identification of cattle, sheep, pigs, chickens, and ducks, and other common meat detection assays aimed at various components (Dalsecco et al. 2018). He et al. applied universal primer multiplex PCR for rapid identification of food pork, beef, mutton, and chicken ingredients (He et al. 2012). Matsunaga assayed six kinds of meat mtDNA with PCR detection at the same time according to the length of DNA fragments to identify the meats present in mixed meat products. In recent years, the development of real-time fluorescent PCR technology has improved the efficiency and sensitivity of species identification in food and made the quantitative traceability of meat content possible (Kesmen et al. 2012).

In particular, the development and application of DNA detection kits for animal food have not been studied in China. The current risk of fake bovine flesh, without a suitable detection regime, has penetrated the market, with all the attendant security risks that this entails. Besides, meat adulteration research is mainly concentrated on cattle, sheep, pigs, chickens, ducks, and other common meats, the development of DNA detection kit in meat products in the detection of mink ingredients in the literature has not yet been reported. The key to the identification of mink elements by molecular genetic marker technology is to extract high-quality DNA. To obtain high-purity DNA, we optimized the SDS alkali modification method and extraction step, and could play an effective role in precipitating protein and polysaccharide. Polyvinylpyrrolidone (PVP), a complex of phenols, was added to the extraction buffer to improve the extraction and purity of nucleic acids. The results showed that the DNA extracted was stable and sufficient in quantity. The design of mtDNA-specific primers is another key used to identify mink-derived components. The specific primers in the kit are *Cyt b* gene sequences with large interspecies differences and are genetically stable. The results showed that the method was both highly specific and accurate. This kit is comparable to the common kit available on the market: its biggest advantage is the collection of DNA extraction and PCR identification takes place in one step. The results showed that the specificity of the kit was 100%. The results of the sensitivity test showed that sample DNA stock solution, when diluted 1000 times, can still be detected; stability test results show that the reactor repeated freezing and thawing treatment 15 times to no adverse effect on the detection effect, and the kit can be stored at –20 °C for 1 year.

The mink component DNA detection kit has good specificity, repeatability, sensitivity, and stability. The kit is highly sensitive and suitable for the detection of fresh meat and processed mixed materials. The mink source components DNA detection kit, for use in meat product quality assays, has a wide range of potential applications.
